# GIPSS: genetically inspired prognostic scoring system for primary myelofibrosis

**DOI:** 10.1038/s41375-018-0107-z

**Published:** 2018-03-23

**Authors:** Ayalew Tefferi, Paola Guglielmelli, Maura Nicolosi, Francesco Mannelli, Mythri Mudireddy, Niccolo Bartalucci, Christy M. Finke, Terra L. Lasho, Curtis A. Hanson, Rhett P. Ketterling, Kebede H. Begna, Naseema Gangat, Animesh Pardanani, Alessandro M. Vannucchi

**Affiliations:** 1 Divisions of Hematology, Departments of Internal Medicine and Laboratory Medicine, Mayo Clinic, Rochester, MN USA; 2 Department of Experimental and Clinical Medicine, CRIMM, Center Research and Innovation of Myeloproliferative Neoplasms, Azienda Ospedaliera Universitaria Careggi, University of Florence, Florence, Italy; 3 Divisions of Hematopathology, Departments of Internal Medicine and Laboratory Medicine, Mayo Clinic, Rochester, MN USA; 4 Divisions of Laboratory Genetics and Genomics, Departments of Internal Medicine and Laboratory Medicine, Mayo Clinic, Rochester, MN USA

## Abstract

International collaborations over the years have produced a series of prognostic models for primary myelofibrosis (PMF), including the recently unveiled mutation-enhanced international prognostic scoring systems for transplant-age patients (MIPSS70 and MIPSS70-plus). In the current study, we considered the feasibility of a genetically inspired prognostic scoring system (GIPSS) that is exclusively based on genetic markers. Among 641 cytogenetically annotated patients with PMF and informative for previously recognized adverse mutations, multivariable analysis identified “VHR” karyotype, “unfavorable” karyotype, absence of type 1/like *CALR* mutation and presence of *ASXL1*,* SRSF2*, or *U2AF1*Q157 mutation, as inter-independent predictors of inferior survival; the respective HRs (95% CI) were 3.1 (2.1–4.3), 2.1 (1.6–2.7), 2.1 (1.6–2.9), 1.8 (1.5–2.3), 2.4 (1.9–3.2), and 2.4 (1.7–3.3). Based on HR-weighted risk points, a four-tiered GIPSS model was devised: low (zero points; *n* = 58), intermediate-1 (1 point; *n* = 260), intermediate-2 (2 points; *n* = 192), and high (≥3 points; *n* = 131); the respective median (5-year) survivals were 26.4 (94%), 8.0 (73%), 4.2 (40%), and 2 (14%) years; the model was internally validated by bootstrapping and its predictive accuracy was shown to be comparable to that of MIPSS70-plus. GIPPS offers a low-complexity prognostic tool for PMF that is solely dependent on genetic risk factors and, thus, forward-looking in its essence.

## Introduction

Primary myelofibrosis (PMF) is an aggressive myeloid malignancy with an estimated median survival of 6 years [1]. Patients with PMF are also at risk for impaired quality of life, as a result of frequent red blood cell transfusion requirement, markedly enlarged spleen and liver, severe constitutional symptoms, cachexia and consequences of portal hypertension, such as ascites, edema, and recurrent gastrointestinal bleeding. Currently employed treatment modalities in PMF (e.g., JAK2 inhibitors, hydroxyurea, immunomodulatory drugs, androgen preparations, corticosteroids, involved-field radiation, and splenectomy), with the exception of allogeneic hematopoietic stem cell transplant (alloSCT), do not modify the natural history of the disease and their value is limited to symptom palliation [2]. Therefore, alloSCT currently remains the treatment of choice in PMF, if the goal of therapy was to prolong life. Unfortunately, alloSCT is associated with a substantial risk of treatment-related mortality and morbidity, and its implementation requires personalized assessment of risk-benefit ratio [3].

Beginning in 2009, international collaborations have produced a series of robust prognostic models in PMF, in order to assist with treatment decision-making and help identify candidates in whom the risk of alloSCT, or other treatment with serious side effects, is justified. The prototype risk models in this regard were initially based on clinically derived variables only [4, 5], while cytogenetic and mutation information was incorporated in the more recent reiterations, including the mutation-enhanced international prognostic scoring systems for transplant-age patients (MIPSS70 and MIPSS70-plus) [6]. The latter included previously acknowledged but further refined clinical risk factors (hemoglobin <10 g/dl, platelets <100 × 10^9^/l, leukocytes >25 × 10^9^/l, circulating blasts ≥2%, constitutional symptoms and grade ≥2 bone marrow fibrosis) and recently highlighted genetic predictors of shortened survival (unfavorable karyotype, absence of *CALR* type 1/like mutation and presence and number of high-molecular risk mutations, including *ASXL1*, *SRSF2*, *EZH2*, and *IDH1/2*); MIPSS70-plus features four risk categories with 5-years survival rates of 7–91% (http://www.mipss70score.it/) [6]. In the current study, we took advantage of the recently revised three-tiered cytogenetic risk stratification in PMF [7], the two-tiered risk stratification according to driver mutational status [8], and the growing list of high risk mutations, including *ASXL1* [9], *SRSF2* [10], and *U2AF1*Q157 [11], in order to recalibrate the inter-independent survival effect of genetic risk factors and provide a new risk model that is exclusively based on mutations and karyotype: genetically inspired prognostic scoring system (GIPSS).

## Methods

The current study was approved by the institutional review boards of the Mayo Clinic, Rochester, MN, USA and the University of Florence, Florence, Italy. All patients provided informed written consent for the study sample collection, as well as permission for its use in research. Inclusion to the current study required availability of archived peripheral blood or bone marrow sample collected at the time of diagnosis (Florence cohort) or first referral (Mayo cohort). Diagnoses of PMF and leukemic transformation were according to the World Health Organization criteria [12]. Cytogenetic analysis and reporting were done according to the International System for Human Cytogenetic Nomenclature criteria [13]. Driver and other mutations were detected by targeted amplicon next generation or direct sequencing, as previously described [6]. Type 1/like and type 2/like *CALR* variant designations were as previously described [14–16]. High-molecular risk mutations included in the current report were selected based on previous reports of prognostic relevance and included *ASXL1*, *SRSF2*, *EZH2*, *IDH1/2*, and *U2AF1* [17, 18]; furthermore, in order to secure optimal sample size and statistical validity, the current study required a minimum of 500 informative cases for a specific mutation to be included in the analysis.

Statistical analyses considered clinical and laboratory parameters obtained at time of diagnosis (University of Florence cohort) or time of diagnosis or first referral (Mayo Clinic cohort), which coincided, in all instances, with time of sample collection for mutation analysis. Differences in the distribution of continuous variables between categories were analyzed by either Mann–Whitney (for comparison of two groups) or Kruskal–Wallis (comparison of three or more groups) test. Patient groups with nominal variables were compared by chi-square test. Overall survival analysis was computed from the date of diagnosis or the first referral (i.e., the date of sample collection) to date of death (uncensored) or last contact (censored). Patients receiving alloSCT were censored at the time of their transplantation. Date of leukemic transformation replaced date of death, as the uncensored variable, for estimating leukemia-free survival. Overall and leukemia-free survival curves were prepared by the Kaplan–Meier method and compared by the log-rank test. Cox proportional hazard regression model was used for multivariable analysis. *P*-values of <0.05 were considered significant. Covariates for the multivariable model were selected based on previous knowledge of their prognostic significance; a step-wise method was used with backward elimination probability threshold of 0.1.

Bootstrap resampling technique, employing 100 bootstrap samplings, was used for internal validation of risk discrimination by the newly developed GIPSS risk model. Additional model validation was accomplished by applying GIPSS to the Mayo and Florence cohorts, separately, as well as to transplant-age patients only (≤70 years old). Relative quality of the GIPSS model, in comparison to the clinically based dynamic international prognostic scoring system (DIPSS) [5] and the more recently published MIPSS70-plus [6] models were estimated by the Akaike information criterion (AIC). In addition, logistic regression was employed to prepare receiver operating characteristic curves and area under the curve (AUC) estimates in order to compare the 10-year mortality prediction performance of GIPSS to both DIPSS and MIPSS70-plus; for the purposes of the particular logistic model, all patients surviving beyond 10 years were censored, while those who died within the particular time frame were uncensored. The JMP® Pro 13.0.0 software from SAS Institute, Cary, NC, USA, was used for all calculations.

## Results

### Baseline patient characteristics

A total of 641 patients with PMF (median age 63 years; 64% males) who were informative for both cytogenetic and mutation information were recruited from the Mayo Clinic, Rochester, MN, USA (*n* = 488) and the University of Florence, Florence, Italy (*n* = 153) (Table 1). Driver mutation distributions were 57% *JAK2*, 19% type 1/like *CALR*, 5% type 2/like *CALR*, 7% *MPL*, and 12% triple negative. DIPSS risk distributions were 13% high, 38% intermediate-2, 33% intermediate-1, and 16% low [5]. MIPSS70-plus risk distributions were very high in 12%, high in 41%, intermediate in 20%, and low in 27% [6]. Cytogenetic risk categories, according to the recently revised system [7], were very high risk (VHR) in 7%, unfavorable in 15% and favorable in 78%. Mutational frequencies were 38% for *ASXL1*, 14% for *SRSF2*, 8% for *U2AF1*Q157, 7% for *EZH2*, and 4% for *IDH1/2*. The frequencies of DIPSS component variables were 41% for age above 65 years, 41% for hemoglobin <10 g/dl, 47% for circulating blasts ≥1%, 14% for leukocyte count >25 × 10^9^/l, and 32% for constitutional symptoms; in addition, 19% displayed platelet count <100 × 10^9^/l and 30% were red cell transfusion dependent.

**Table 1:** Clinical and laboratory characteristics of 641 patients with primary myelofibrosis stratified by center of referral: Mayo Clinic, Rochester, MN, USA vs. University of Florence, Florence, Italy

Variables	All patients (*n* = 641)^a^	Mayo Clinic cohort (*n* = 488)	University of Florence cohort (*n* = 153)	*P*-value
Age in years; median (range)	63 (19–89)	63 (22–87)	62 (19–89)	0.2
Age >65 years; *n*(%)	263 (41)	202 (41)	61(40)	0.7
Males (%)	411 (64)	310 (64)	101 (66)	0.6
Hemoglobin <10 g/dl; *n* (%)	260 (41)	217 (45)	43 (28)	<**0.001**
Transfusion requiring; *n* (%)	191 (30)	156 (32)	35 (23)	**0.03**
Leukocytes, x10^9^/l; median (range)	9 (1–219)	9 (1–219)	9 (2–150)	0.7
Leukocytes >25 × 10^9^/l;*n* (%)	89 (14)	71(15)	18 (13)	0.6
Platelets <100 × 10^9^/l;*n* (%)	122 (19)	104 (21)	18 (13)	**0.02**
Circulating blasts ≥1%; *n* (%)	297 (47)	262 (54)	35 (24)	<**0.001**
Circulating blasts ≥2%; *n* (%)	173 (27)	148 (30)	25 (16)	<**0.001**
Constitutional symptoms; *n* (%)	208 (32)	161 (33)	47 (31)	0.6
*DIPSS* ^b^ *risk distribution*				**<0.001**
High	83 (13)	50 (10)	33 (22)	
Intermediate-2	242 (38)	188 (39)	54 (35)	
Intermediate-1	214 (33)	176 (36)	38 (25)	
Low	102 (16)	74 (15)	28 (18)	
*Driver mutations*				**0.03**
JAK2; *n* (%)	368 (57)	288 (59)	80 (53)	
CALR type 1/like; *n* (%)	123 (19)	99 (20)	24 (16)	
CALR type 2/like; *n* (%)	32 (5)	19 (4)	13 (8)	
MPL;*n* (%)	46 (7)	33 (7)	13 (8)	
Triple negative; *n* (%)	72 (12)	49 (10)	23 (15)	
*Revised cytogenetic risk distribution* ^c^				*0.2*
Very high risk;* n* (%)	43 (7)	32 (7)	11 (7)	
Unfavorable; *n* (%)	94 (15)	78 (16)	16 (11)	
Favorable;* n* (%)	504 (78)	378 (77)	126 (82)	
*ASXL1*-mutated;*n* (%)	242 (38)	188 (39)	54 (35)	0.5
*SRSF2*-mutated; *n* (%)	89 (14)	70 (14)	19 (12)	0.5
*U2AF1Q157*-mutated; *n*(%)	50 (8)	46 (9)	4 (3)	**0.006**
*EZH2*-mutated; *n*(%)	37 (7)	16 (4)	21 (14)	**<0.001**
*IDH1/2*-mutated; *n* (%)	23 (4)	20 (5)	3 (2)	0.1
*MIPSS70-plus risk distribution* ^d^				***0.005***
Very high; *n* (%)	76 (12)	58 (12)	18 (12)	
High; *n* (%)	263 (41)	216 (44)	47 (31)	
Intermediate; *n* (%)	125 (20)	95 (20)	30 (19)	
Low; *n* (%)	177 (27)	119 (24)	58 (38)	

The values in bold indicate a significant *P*-value (<0.05)

*ASXL1* additional sex combs like 1, *SRSF2* Serine/arginine-rich splicing factor 2, *U2AF1 U2*small nuclear RNA auxiliary factor 1*, EZH2* enhancer of zeste homolog 2, *IDH1/2* isocitrate dehydrogenase 1/2, *JAK2* Janus kinase 2,*CALR* calreticulin, *MPL* myeloproliferative leukemia virus oncogene

^a^ In most instances, including all GIPSS-relevant variables, information was available in all 641 patients. In all instances of genetic risk factor analysis, a minimum of 500 informative cases was required and missing information did not exceed 10%

^b^ DIPSS, Dynamic International Prognostic Scoring System uses five independent predictors of inferior survival: age > 65 years, hemoglobin <10 g/dl, leukocytes >25 × 10^9^/L, circulating blasts ≥1% and constitutional symptoms (reference in the text)

^c^ Revised cytogenetic risk stratification: “very high risk (VHR)”—single/multiple abnormalities of −7, i(17q), inv(3)/3q21, 12p−/12p11.2, 11q−/11q23, +21, or other autosomal trisomies, not including +8/+9; “favorable”—normal karyotype or sole abnormalities of 13q−, +9, 20q−, chromosome 1 translocation/duplication or sex chromosome abnormality including—Y; “unfavorable”—all other abnormalities (reference in the text)

^d^ MIPSS70-plus, Mutation-Enhanced International Prognostic Score System for transplant-age patients uses: hemoglobin <10 g/dl, leukocytes >25 × 10^9^/L, platelets <100 × 10^9^/L, circulating blasts ≥2%, constitutional symptoms, absence of *CALR* type 1 mutation, presence of high-molecular risk mutation (e.g., *ASXL1*, *EZH2*, *SRSF2*, *IDH1/2*), presence of two or more high-molecular risk mutations and a two-tiered revised cytogenetic risk stratification where very high risk and unfavorable karyotype are grouped together as “unfavorable” (reference in the text)

Tables 1 and 2 provide additional information on distribution of clinical and laboratory variables stratified by the Mayo vs. Florence patient cohorts (Table 1) and the revised cytogenetic risk stratification (Table 2). Significant differences in the characteristics of patients from the Mayo Clinic vs. those from the University of Florence were mostly attributed to differences in time point of evaluation, as mentioned earlier in the Methods section, and best reflected in their MIPSS70-plus risk distribution (Table 1). Patients with VHR or unfavorable karyotype were more likely to display adverse clinical characteristics, including severe anemia, platelet count <100 × 10^9^/l, increased circulating blast count and accordingly clustered with higher risk DIPSS categories; high risk molecular mutations were also more prevalent in patients with VHR karyotype (Table 2).

**Table 2:** Clinical and laboratory characteristics of 641 patients with primary myelofibrosis stratified by the revised cytogenetic risk model^a^

Variables	All patients (*n* = 641)^b^	Very high risk karyotype (*n* = 43)	Unfavorable karyotype (*n* = 94)	Favorable karyotype (*n* = 504)	*P*-value
Age in years; median (range)	63 (19–89)	65 (46–87)	64 (38–81)	62 (19–89)	**0.02**
Age >65 years; *n* (%)	263 (41)	22 (51)	38 (40)	203 (40)	0.4
Males (%)	411 (64)	26 (60)	67 (71)	318 (63)	0.3
Hemoglobin <10 g/dl; *n* (%)	260 (41)	28 (65)	42 (45)	190 (38)	**0.001**
Transfusion requiring; *n* (%)	191 (30)	25 (58)	30 (32)	136 (27)	**<0.001**
Leukocytes, x10^9^/l; median (range)	9 (1–219)	10 (2–75)	8 (1.4–219)	9.2 (1–176)	0.5
Leukocytes >25 × 10^9^/l; *n* (%)	89 (14)	9 (23)	14 (15)	66 (13)	0.2
Platelets, x10^9^/l; median (range)	237 (10–2466)	123.5 (11–1000)	154 (10–2282)	261 (12–2466)	**<0.001**
Platelets <100 × 10^9^/l; *n* (%)	122 (19)	18 (45)	24 (26)	80 (16)	**<0.001**
Circulating blasts ≥1%; *n* (%)	297 (47)	29 (71)	49 (54)	219 (44)	**0.001**
Circulating blasts ≥2%; *n* (%)	173 (27)	23 (53)	29 (31)	121 (24)	**<0.001**
Constitutional symptoms; *n* (%)	208 (32)	19 (44)	36 (38)	153 (30)	0.07
*DIPSS* ^c^ * risk distribution*					***<0.001***
High; *n* (%)	83 (13)	14 (33)	14 (15)	55 (11)	
Intermediate-2; *n* (%)	242 (38)	24 (56)	36 (38)	182 (36)	
Intermediate-1 *n* (%)	214 (33)	3 (7)	35 (37)	176 (35)	
Low; *n* (%)	102 (16)	2 (4)	9 (10)	91 (18)	
*Driver mutations*					*0.14*
JAK2; *n* (%)	368 (57)	21 (49)	56 (60)	291 (57)	
CALR type 1/like; *n* (%)	123 (19)	6 (14)	23 (24)	94 (19)	
CALR type 2/like; *n* (%)	32 (5)	3 (7)	2 (2)	27 (5)	
MPL; *n* (%)	46 (7)	3 (7)	4 (4)	39 (8)	
Triple negative; *n* (%)	72 (12)	10 (23)	9 (10)	53 (11)	
*ASXL1*-mutated; *n* (%)	242 (38)	24 (56)	35 (37)	183 (36)	**0.04**
*SRSF2*-mutated; *n* (%)	89 (14)	12 (28)	9 (10)	68 (13)	**0.01**
*U2AF1Q157*-mutated; *n* (%)	50 (8)	2 (5)	7 (7)	41 (8)	0.7
*EZH2*-mutated; *n* (%)	37 (7)	3 (8)	4 (5)	30 (7)	0.7
*IDH1/2*-mutated; *n* (%)	23 (4)	2 (5)	3 (4)	18 (4)	0.9
*MIPSS70-plus* ^d^ * risk distribution*					***<0.001***
Very high; *n* (%)	76 (12)	33 (77)	37 (39)	6 (1)	
High; *n* (%)	263 (41)	10 (23)	53 (57)	200 (40)	
Intermediate; *n* (%)	125 (20)	0 (0)	4 (4)	121 (24)	
Low; *n* (%)	177 (27)	0 (0)	0 (0)	177 (35)	

The values in bold indicate a significant *P*-value (<0.05)

*ASXL1* additional sex combs like 1, *SRSF2* serine/arginine-rich splicing factor 2, *U2AF1 U2*small nuclear RNA auxiliary factor 1*, EZH2* enhancer of zeste homolog 2, *IDH1/2* isocitrate dehydrogenase 1/2, *JAK2* Janus kinase 2, *CALR* calreticulin, *MPL* myeloproliferative leukemia virus oncogene

^a^ Revised cytogenetic risk stratification: “very high risk (VHR)”—single/multiple abnormalities of −7, i(17q), inv(3)/3q21, 12p−/12p11.2, 11q−/11q23, +21, or other autosomal trisomies, not including +8/+9; “favorable”—normal karyotype or sole abnormalities of 13q−, +9, 20q−, chromosome 1 translocation/duplication or sex chromosome abnormality including—Y; “unfavorable”—all other abnormalities (reference in the text)

^b^ In most instances, including all GIPSS-relevant variables, information was available in all 641 patients. In all instances of genetic risk factor analysis, a minimum of 500 informative cases was required and missing information did not exceed 10%

^c^ DIPSS, Dynamic International Prognostic Scoring System uses five independent predictors of inferior survival: age >65 years, hemoglobin <10 g/dl, leukocytes >25 × 10^9^/L, circulating blasts ≥1% and constitutional symptoms (reference in the text)

^d^ MIPSS70-plus, Mutation-Enhanced International Prognostic Score System for transplant-age patients uses: hemoglobin <10 g/dl, leukocytes >25 × 10^9^/L, platelets <100 × 10^9^/L, circulating blasts ≥2%, constitutional symptoms, absence of *CALR* type 1 mutation, presence of high-molecular risk mutation (e.g., *ASXL1*, *EZH2*, *SRSF2*, *IDH1/2*), presence of two or more high-molecular risk mutations and a two-tiered revised cytogenetic risk stratification where very high risk and unfavorable karyotype are grouped together as “unfavorable” (reference in the text)

### Univariate and multivariable analyses of genetic risk factors for overall survival and their interaction with DIPSS

After a median follow-up of 3.9 years (5.8 years for living patients), 380 (59%) deaths, 73 (11%) leukemic transformations, and 45 (7%) stem cell transplants were recorded. In univariate analysis of overall survival, the revised cytogenetic risk stratification, absence of type 1/like *CALR* mutation, presence of *ASXL1*, *SRSF2*, or *U2AF1*Q157 mutations were significantly associated with inferior survival (*p* < 0.001 in all instances; Table 3); significance was not apparent for *IDH1/2* (*p* = 0.07) or *EZH2* mutations (*p* = 0.2). In multivariable analysis restricted to genetic risk factors, significance was retained for VHR karyotype (HR 3.1; 95% CI 2.1–4.3), unfavorable karyotype (HR 2.1, 95% CI 1.6–2.7), absence of type 1/like *CALR* mutation (HR 2.1, 95% CI 1.6–2.9) or presence of *ASXL1* (HR 1.8, 95% CI 1.5–2.3), *SRSF2* (HR 2.4, 95% CI 1.9–3.2), or *U2AF1*Q157 (HR 2.4, 95% CI 1.7–3.3) mutations; *EZH2* and *IDH1/2* mutations remained not significant during multivariable analysis. The addition of DIPSS risk scores in the multivariable model did not undermine the independent prognostic effect of the aforementioned mutations while it confirmed persistence of residual significance from the clinically derived DIPSS (Table 3); HRs (95% CI values) in DIPSS-inclusive multivariable analysis were 2.5 (1.7–3.5) for VHR karyotype, 1.9 (1.4–2.5) for unfavorable karyotype, 2.0 (1.5–2.8) for absence of type 1/like *CALR* mutation, 1.6 (1.3–2.0) for *ASXL1*, 2.2 (1.7–2.8) for *SRSF2* and 1.9 (1.4–2.7) for *U2AF1*Q157 mutations and 4.6 (2.8–7.4) for DIPSS high, 4.2 (2.7–6.5) for DIPSS intermediate-2, 2.6 (1.7–4.1) for DIPSS intermediate-1 risk categories (Table 3).

**Table 3:** Univariate and multivariable analysis of genetic risk factors for overall and leukemia-free survival among 641 patients with primary myelofibrosis

Overall survival		
Variables	Univariate analysis *P*-value (HR, 95% CI)	Multivariable analysis *P*-value (HR, 95% CI)
*Revised cytogenetic risk model* ^a^	***<0.001***	***<0.001***
Very high risk karyotype	< **0.001 (3.6, 2.6**–**5.1)**	< **0.001 (2.5, 1.7**–**3.5)**
Unfavorable karyotype	**<0.001 (1.9, 1.4**–**2.5)**	**<0.001 (1.9, 1.4**–**2.5)**
Favorable karyotype	Reference	Reference
*ASXL1*-mutated	**<0.001 (2.1, 1.7**–**2.6)**	**<0.001 (1.6, 1.3**–**2)**
*SRSF2*-mutated	**<0.001 (2.6, 1.9**–**3.3)**	**<0.001 (2.2, 1.7**–**2.**
*U2AF1*Q157-mutated	**<0.001 (2.6, 1.8**–**3.6)**	**0.002 (1.9, 1.4**–**2.7)**
*EZH2*-mutated	0.2 (1.3, 0.8–1.9)	
*IDH1* or *IDH2*-mutated	0.07 (1.6, 0.9–2.6)	
*Driver mutational status*	***<0.001***	***<0.001***
* JAK2*	<**0.001 (2.3, 1.7**–**3.1)**	<**0.001 (2.2, 1.6**–**3.0)**
* MPL*	**0.003 (2.3, 1.5**–**3.6)**	**0.03 (1.6, 1.1**–**2.6)**
Triple negative	<**0.001 (2.9, 1.9**–**4.3)**	<**0.001 (2.3, 1.5**–**3.5)**
* Type 2/like CALR*	0.1 (1.6, 0.9–2.7)	
* Type 1/like CALR*	**Reference**	**Reference**
* Type 1/like CALR* absent	**<0.001 (2.3, 1.7**–**3.1)**	**<0.001 (2.0, 1.5**–**2.8)**
*DIPSS* ^b^	***<0.001***	*<* ***0.001***
High	<**0.001 (8.3, 5.2**–**13.3)**	<**0.001 (4.6, 2.8**–**7.4)**
Intermediate-2	**<0.001 (5.6, 3.6**–**8.6)**	<**0.001 (4.2, 2.7**–**6.5)**
Intermediate-1	<**0.001 (2.9, 1.9**–**4.5)**	<**0.001 (2.6, 1.7**–**4.1)**
Low	Reference	Reference
	Leukemia-free survival	
Variables	Univariate analysis *P* (HR, 95%CI)	Multivariable analysis *P* (HR, 95%CI)
*Revised cytogenetic risk model*	***<0.001***	***0.002***
Very high risk karyotype	<**0.001 (4.6, 2.3**–**9.5)**	**0.04 (2.4, 1.02**–**5.5)**
Unfavorable karyotype	**0.005 (2.3, 1.3**–**4.1)**	**0.0009 (2.7, 1.5**–**4.9)**
Favorable karyotype	Reference	Reference
*ASXL1*-mutated	**<0.001 (2.6, 1.6**–**4.1)**	**0.004 (2.1, 1.3**–**3.4)**
*SRSF2*-mutated	**<0.001 (3.9, 2.3**–**6.7)**	**<0.001 (4.3, 2.5**–**7.5)**
*U2AF1*Q157*-*mutated	0.8 (1.1, 0.4–3.1)	
*EZH2*-mutated	0.06 (2.0, 0.9–4.2)	
*IDH1* or *IDH2-*mutated	**0.005 (3.1, 1.4**–**6.7)**	
*Driver mutational status*	***0.04***	
* JAK2*	0.4 (1.3, 0.7–2.4)	
* MPL*	0.4 (1.5, 0.6–4.0)	
Triple negative	**0.005 (2.9, 1.4**–**6.2)**	
* Type 2/like CALR*	0.1 (0.9, 0.3–3.5)	
* Type 1/like CALR*	Reference	
*Type 1/like CALR* absent	0.2 (1.5, 0.8–2.6)	
Platelets <100 × 10^9^/l	**0.007 (2.5, 1.5**–**4.2)**	**0.002 (2.3, 1.3**–**4.0)**
Circulating blasts ≥2%	**<0.001 (3.3, 2.0**–**5.3)**	**0.001 (2.6, 1.6**–**4.3)**
*DIPSS* ^b^	***0.005***	
High	**0.002 (7.2, 2.5**–**20.4)**	
Intermediate-2	**0.001 (4.9, 1.9**–**12.5)**	
Intermediate-1	**0.04** (**2.7, 1.03**–**7.3)**	
Low		

The values in bold indicate a significant *P*-value (<0.05)

*ASXL1* additional sex combs like 1, *SRSF2* serine/arginine-rich splicing factor 2, *U2AF1 U2* small nuclear RNA auxiliary factor 1*, EZH2* enhancer of zeste homolog 2, *IDH1/2* isocitrate dehydrogenase 1/2, *JAK2* Janus kinase 2,* CALR* calreticulin, *MPL* myeloproliferative leukemia virus oncogene

^a^ Revised cytogenetic risk stratification: “very high risk (VHR)”—single/multiple abnormalities of −7, i(17q), inv(3)/3q21, 12p−/12p11.2, 11q−/11q23, +21, or other autosomal trisomies, not including +8/+9; “favorable”—normal karyotype or sole abnormalities of 13q−, +9, 20q−, chromosome 1 translocation/duplication or sex chromosome abnormality including—Y; “unfavorable”—all other abnormalities (reference in the text)

^b^ DIPSS, Dynamic International Prognostic Scoring System uses five independent predictors of inferior survival: age >65 years, hemoglobin <10 g/dL, leukocytes >25 × 109/L, circulating blasts ≥1% and constitutional symptoms (reference in the text)

### Univariate and multivariable analysis of genetic risk factors for leukemia-free survival and their interaction with other risk factors for leukemic transformation

In univariate analysis of genetic risk factors, leukemia-free survival was predicted by karyotype (*p* < 0.001), *SRSF2* mutation (*p* < 0.001), *ASXL1* mutation (*p* < 0.001), *IDH1/2* mutations (*p* = 0.005), and triple negative mutational status (*p* = 0.005) (Table 3); *U2AF1*Q157 mutations had no significance (*p* = 0.8), while *EZH2* mutations displayed borderline significance (*p* = 0.06). In multivariable analysis that also included other risk factors for leukemic transformation (Table 3), karyotype (HR 2.4, 95% CI 1.02–5.5 for VHR karyotype and HR 2.7, 95% CI 1.5–4.9 for unfavorable karyotype), *SRSF2* mutations (HR 4.3, 95% CI 2.5–7.5), *ASXL1* mutations (HR 2.1, 95% CI 1.3–3.4), platelet count <100 × 10^9^/l (HR 2.3, 95% CI 1.3–4.0), and circulating blasts ≥2% (HR 2.6, 95% CI 2.6, 95% CI 1.6–4.3) remained significant (Table 3).

### Development of a new risk model (GIPSS) that is exclusively based on genetic risk factors

**Fig. 1:**
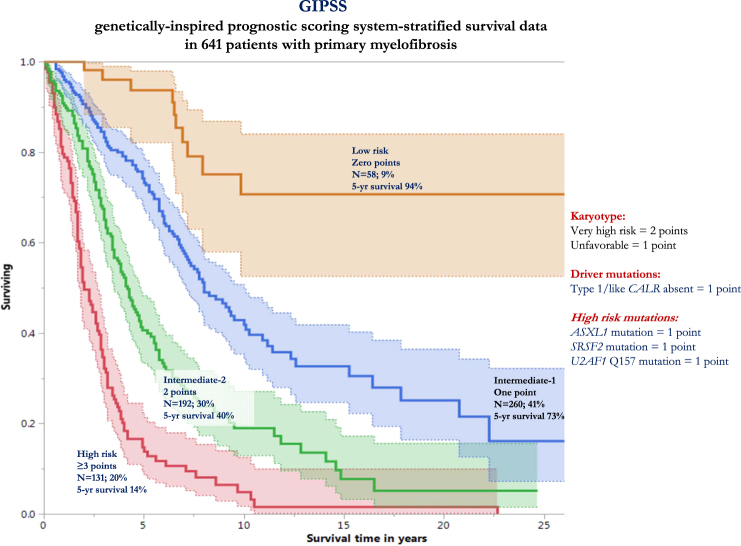
Genetically inspired prognostic scoring system (GIPSS)-stratified survival data in 641 patients with primary myelofibrosis. Median survivals were 2 years for GIPSS high risk, 4.2 years for intermediate-2, 8 years for intermediate-1, and 26.4 years for low risk. The number of patients at risk for high, intermediate-2, intermediate-1, and low risk GIPSS at 5 years were 15, 61, 150, and 41; at 10 years 4, 15, 41, and 17; and at 15 years 2, 5, 16, and 10

**Fig. 2:**
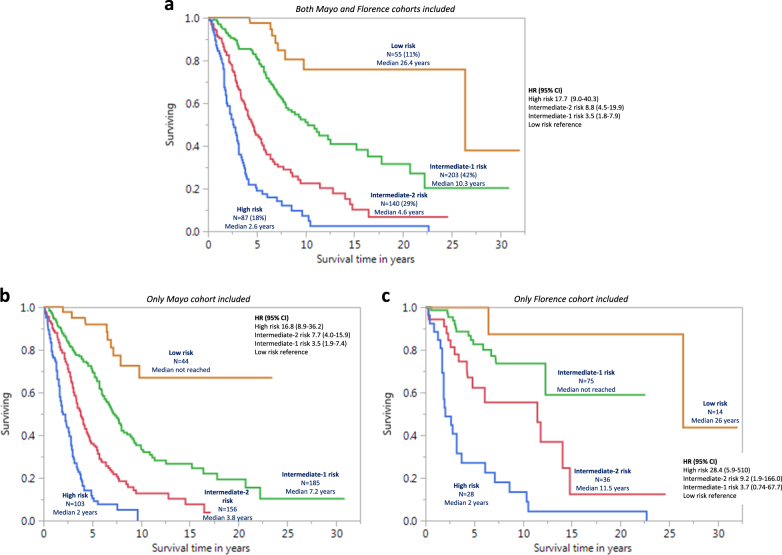
**a** Genetically inspired prognostic scoring system (GIPSS)-stratified survival data in 485 patients with primary myelofibrosis and age 70 years or younger, including both Mayo and Florence cohorts. **b** GIPSS-stratified survival data in 488 Mayo Clinic patients with primary myelofibrosis, including Mayo cohort only. **c** GIPSS-stratified survival data in 153 Italian patients with primary myelofibrosis, including Florence cohort only

Risk points were allocated to each one of the above-mentioned inter-independent genetic risk factors based on HRs derived from multivariable analysis of genetic risk factors (see above): two points for VHR karyotype (HR 3.1) and one point each for unfavorable karyotype (HR 2.1), absence of type 1/like *CALR* mutation (HR 2.1) or presence of *ASXL1* (HR 1.8), *SRSF2* (HR 2.4) or *U2AF1*Q157 (HR 2.4) mutations. The sum of risk points for each patient was calculated and used to develop a four-tiered GIPSS: low risk with zero points (*n* = 58), intermediate-1 risk with one point (*n* = 260), intermediate-2 risk with two points (*n* = 192), and high risk with three or more points (*n* = 131); the respective median (5-year) survival rates were 26.4 years (94%), 8.0 years (73%), 4.2 years (40%), and 2 years (14%) years (Fig. 1); HRs (95% CI), using the low risk group as the reference, were 15.8 (8.8–31.3) for high risk, 7.1 (4.0–14.0) for intermediate-2 risk, and 3.2 (1.8–6.4) for intermediate-1 risk; the bootstrap 95% confidence limits were 7.6–35.2 for high risk, 3.4–12.7 for intermediate-2 risk, and 1.6–6.2 for intermediate-1 risk. Additional inter-risk group comparisons included HRs (95% CI) of 4.9 (3.7–6.3) for high vs. intermediate-1 risk (bootstrap 95% confidence limit 3.2–6.5), 2.2 (1.7–2.9) for high vs. intermediate-2 risk (bootstrap 95% confidence limit 1.6–3.0) and 2.2 (1.7–2.8) for intermediate-2 vs. intermediate-1 risk (bootstrap 95% confidence limit 1.8–2.8). Additional model validation was accomplished by applying GIPSS to the Mayo (*n* = 488) and Florence (*n* = 153) patient cohorts separately (Fig. 2b, c), as well as to transplant-age (age ≤70 years) patients (*n* = 485; Fig. 2a); the lack of significant difference between low and intermediate-1 risk GIPSS groups in the Italian patient cohort was attributed to inadequate sample size.

**Fig. 3:**
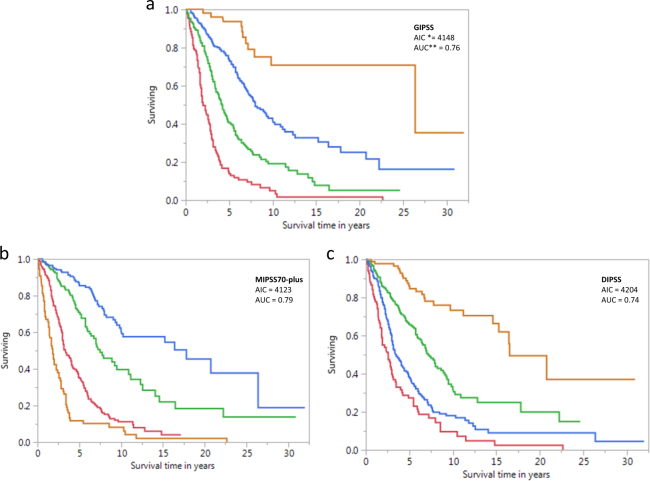
Comparison of survival data in 641 patients with primary myelofibrosis stratified by genetically inspired prognostic scoring system (GIPSS; Fig. 3a), mutation-enhanced international prognostic scoring system (MIPSS70-plus; Fig. 3b), or dynamic international prognostic scoring system (DIPSS; Fig. 2c). *AIC Akaike information criterion, **AUC area under the curve

**Fig. 4:**
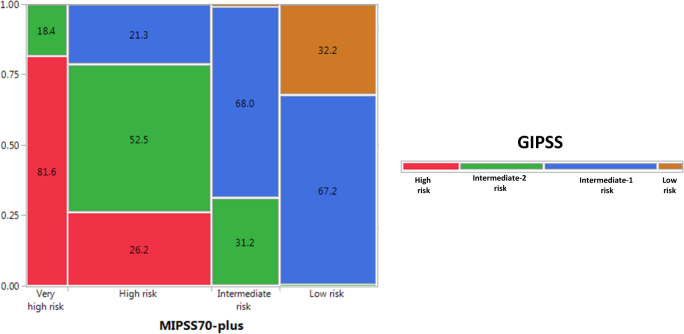
Risk distribution among 641 patients with primary myelofibrosis according to GIPSS (genetically inspired prognostic scoring system) and MIPSS70-plus (mutation-enhanced international prognostic system including karyotype) (numbers in cells indicate percentages)

Figure 3 displays survival curves from the current dataset stratified by GIPSS (Fig. 3a), MIPSS70-plus (Fig. 3b), and DIPSS (Fig. 3c). AIC and AUC estimates were comparable between GIPSS (AIC 4148, AUC 0.76) and MIPSS70-plus (AIC 4123, AUC 0.79) and both appeared to be superior to those of DIPSS (AIC 4204, AUC 0.74). Furthermore, as illustrated in Fig. 4, there was significant alignment of risk distribution between GIPSS and MIPSS70-plus, especially for “low” and “high” risk patients. In other words, a patient with GIPSS “high” risk disease is most likely to also be in the MIPSS70-plus “high” or “very high” risk category whereas a patient with GIPSS “low” risk disease is almost certain to be in the MIPSS70-plus “low” risk category as well (Fig. 4). In other words, additional prognostic information from MIPSS70-plus might not be necessary in GIPSS “high” or “low” risk disease categories. On the other hand, a patient with GIPSS “intermediate-1” risk disease might be reclassified as MIPSS70-plus low, intermediate or high risk disease and one with GIPSS intermediate-2 risk disease as MIPSS70-plus very high, high or intermediate risk disease (Fig. 4). Finally, GIPSS was shown to be effective in also predicting leukemia-free survival; HRs (95% CI) were 16.6 (4.8–104.1) for VHR, 7.0 (2.1–43.8) for high risk and 3.0 (0.9–18.6) for low risk GIPSS categories.

## Discussion

At present, the two main clinically derived risk models in PMF, IPSS [4], and DIPSS [5], remain useful for routine patient management. However, higher level care requires additional biologic information that not only refines prognostication but might also guide the implementation of targeted therapy [19]. Towards that end, cytogenetic information was first incorporated into the DIPSS model, resulting in DIPSS-plus [20], and more recently both cytogenetic and mutation information were utilized in the development of MIPSS70-plus [6]. The latter was designed with transplant-age patients (age ≤70 years) in mind and was based on four clinical (hemoglobin <10 g/dl, leukocyte count >25 × 10^9^/l, circulating blasts ≥2% and constitutional symptoms) and three genetic risk components (karyotype, driver mutational status and high risk mutations). Since the publication of MIPSS70-plus in December 2017 [6], we have further refined cytogenetic risk stratification in PMF [7] and also identified *U2AF1*Q157 mutation as a new independent risk factor for overall survival [11], thus providing the opportunity to develop a new risk model that is exclusively based on genetic risk factors.

GIPSS represents the first step in our aspiration to fully replace clinical variables with genetic markers, for prediction of survival in PMF. Our working hypothesis, in this regard, considers clinical phenotype in PMF as a surrogate for currently known and unknown underlying genetic lesions. In the current study, the inter-independent prognostic relevance of previously recognized adverse mutations in PMF was vetted by multivariable analysis that also included driver mutational status and the revised cytogenetic risk stratification; accordingly the study confirmed the independent prognostic relevance of VHR karyotype, unfavorable karyotype and certain mutations including the prognostically favorable type 1/like *CALR* mutation and the prognostically unfavorable *ASXL1*, *SRSF2*, and *U2AF1*Q157 mutations; the respective frequencies of these prognostic biomarkers, at time of patient referral to a tertiary care center were approximately 8, 19, 15, 38, 14, and 9% [11, 17]. As underlined in the Methods section, the current study required a minimum of 500 informative cases for a specific mutation to be included in the analysis. Accordingly, the additional prognostic contribution of other prognostically relevant but less frequent mutations, such as *LNK*, *RUNX1*, and *CBL* was not addressed in the current report [18]. It should also be noted that the lack of multivariable significance for *EZH2* or *IDH1/IDH2* mutations, in the current study, should not be regarded as being definitive. In other words, GIPSS should not be considered as a finished product but rather a template for incorporating additional genetic information, as it becomes available. In this regard, it is crucial to recognize the important prognostic interaction between karyotype and mutations and the prospect of considering additional mutations in future genetic risk models requires clear demonstration of their karyotype-independent prognostic value; for example, the presence of high risk mutations imparts little to no additional prognostic effect in patients with VHR karyotype whereas their absence provides additional comfort in asserting the excellent prognosis associated with favorable karyotype [7].

GIPSS offers a low-complexity and practical risk model for PMF that is based exclusively on karyotype and a limited number of mutations, including *ASXL1*, *SRSF2*, *U2AF1*, and *CALR*. Application of GIPSS requires familiarity with the recently revised three-tiered cytogenetic risk stratification for PMF [7], as well as recognition of the prognostic distinction between different *CALR* and *U2AF1* mutation variants [8, 11, 14]. In regards to the former, the new cytogenetic risk categories include “favorable” (normal karyotype or sole abnormalities of 20q−, 13q−, +9, chromosome 1 translocation/duplication or sex chromosome abnormality including—Y), “VHR” (single or multiple abnormalities of −7, inv(3), i(17q), 12p−, 11q−, and autosomal trisomies other than +8 or +9) and “unfavorable” (all other abnormalities) karyotype [7]. Assessment of *ASXL1* and *SRSF2* mutations is uncomplicated since one is simply required to document their presence or absence; we have recently reported that the type of *ASXL1* mutation did not affect its prognostic relevance [9]. In contrast, determining the type of mutation is prognostically critical for both *U2AF1* and *CALR*. *U2AF1* mutations in PMF involve either the Q157 or S34 amino acid positions, but only those affecting the Q157 residue (i.e., Q157P and Q157R) are prognostically relevant [11]. Similarly, *CALR* mutations in PMF come in two types: type 1/like and type 2/like [14]. Type 1 *CALR* mutations constitutes a 52-bp deletion (p.L367fs*46) and type 2 a 5-bp TTGTC insertion (p.K385fs*47). Non-type 1 or type 2 *CALR* mutations are categorized as type 1/like and type 2/like variants, based on structural similarities (alpha helix propensity) to the corresponding classical mutants [14, 16]. It is now well-established that the favorable survival effect of *CALR* mutations in PMF is fully attributed to only its type 1/like variant [14, 15, 21].

**Fig. 5:**
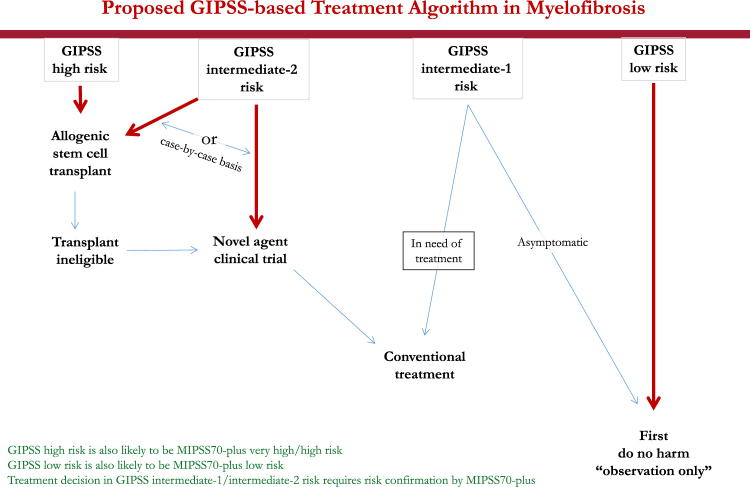
Proposed treatment decision tree, including timing of allogeneic stem cell transplant, based on GIPSS (genetically inspired prognostic scoring system)-based risk stratification. It is underscored that the proposed algorithm is provided in order to illustrate the potential value of GIPSS in clinical practice, and not as a definitive treatment guideline, which requires additional validation

Taken together, one can envision a step-wise prognostication approach in PMF that starts with the simpler GIPSS model that is based on karyotype and mutations only, and reliably select candidates for alloSCT (GIPSS high risk disease) or long-term observation with little or no therapeutic intervention (GIPSS low risk disease) (Fig. 5). In other words, for the purposes of major therapeutic decisions, additional prognostic information from MIPSS70-plus or other clinically derived prognostic models (e.g., IPSS and DIPSS) might not be necessary for GIPSS “high” or GIPSS “low” risk patients (Figs. 4 and 5). On the other hand, we favor more comprehensive risk scoring for prognostication in GIPSS intermediate-1 or intermediate-2 risk disease, which is currently provided by MIPSS70-plus (http://www.mipss70score.it/) [6]; for example, as outlined in Fig. 4, approximately 20% of patients with GIPSS intermediate-1 risk disease are reclassified as high risk, according to MIPSS70-plus, which is a treatment-relevant change in risk status; whether or not the outcome of this particular group of patients is more in line with their GIPSS or MIPSS70-plus risk level requires further investigation. Regardless, using conventional statistical tools (e.g., AIC and AUC), we were able to demonstrate the non-inferiority of GIPSS, compared to MIPSS70-plus and other prognostic models for PMF, in its discrimination ability and prediction accuracy (Fig. 3). The fact that clinical variables in PMF currently continue to display mutation- and karyotype-independent prognostic significance is more a reflection of our truncated knowledge regarding the genetic makeup of the underlying clonal process, rather than the quality of their performance. Accordingly, it is our full intention to continue recruiting additional mutations of prognostic relevance in PMF and further limit prognostic reliance on clinical variables.

## References

[1] Tefferi A, Guglielmelli P, Larson DR, Finke C, Wassie EA, Pieri L (2014). Long-term survival and blast transformation in molecularly annotated essential thrombocythemia, polycythemia vera, and myelofibrosis. Blood.

[2] Cervantes F, Pereira A (2017). Does ruxolitinib prolong the survival of patients with myelofibrosis?. Blood.

[3] Farhadfar N, Cerquozzi S, Patnaik M, Tefferi A (2016). Allogeneic hematopoietic stem-cell transplantation for myelofibrosis: a practical review. J Oncol Pract.

[4] Cervantes F, Dupriez B, Pereira A, Passamonti F, Reilly JT, Morra E (2009). New prognostic scoring system for primary myelofibrosis based on a study of the International Working Group for Myelofibrosis Research and Treatment. Blood.

[5] Passamonti F, Cervantes F, Vannucchi AM, Morra E, Rumi E, Pereira A (2010). A dynamic prognostic model to predict survival in primary myelofibrosis: a study by the IWG-MRT (International Working Group for Myeloproliferative Neoplasms Research and Treatment). Blood.

[6] Guglielmelli P, Lasho TL, Rotunno G, Mudireddy M, Mannarelli C, Nicolosi M (2018). MIPSS70: Mutation-Enhanced International Prognostic Score System for transplantation-age patients with primary myelofibrosis. J Clin Oncol.

[7] Tefferi A, Nicolosi M, Mudireddy M, Lasho TL, Gangat N, Begna KH, et al. Revised cytogenetic risk stratification in primary myelofibrosis: analysis based on 1002 informative patients. Leukemia. 2018. 10.1038/s41375-018-0018-z (ISSN: 1476-5551). 10.1038/s41375-018-0018-z PMC5940654 29472717

[8] Tefferi A, Nicolosi M, Mudireddy M, Szuber N, Finke CM, Lasho TL, et al. Driver mutations and prognosis in primary myelofibrosis: Mayo-Careggi MPN alliance study of 1,095 patients. Am J Hematol. 2017. 10.1002/ajh.24978. 10.1002/ajh.24978 29164670

[9] Tefferi A, Lasho TL, Finke C, Gangat N, Hanson CA, Ketterling RP, et al. Prognostic significance of ASXL1 mutation types and allele burden in myelofibrosis. Leukemia*.*2017. 10.1038/leu.2017.318. 10.1038/leu.2017.318 29089644

[10] Tefferi A, Lasho TL, Hanson CA, Ketterling RP, Gangat N, Pardanani A. Screening for ASXL1 and SRSF2 mutations is imperative for treatment decision-making in otherwise low or intermediate-1 risk patients with myelofibrosis. Br J Haematol. 2017. 10.1111/bjh.15010. 10.1111/bjh.15010 29171022

[11] Tefferi A, Finke CM, Lasho TL, Hanson CA, Ketterling RP, Gangat N, et al. *U2AF1* mutation types in primary myelofibrosis: phenotypic and prognostic distinctions. Leukemia. 2018, in press. 10.1038/s41375-018-0078-0 PMC6170397 29535431

[12] Vardiman JW, Thiele J, Arber DA, Brunning RD, Borowitz MJ, Porwit A (2009). The2008 revision of the World Health Organization (WHO) classification of myeloid neoplasms and acute leukemia: rationale and important changes. Blood.

[13] McGowan-Jordan J, Simons A, Schmid M. An International System for Human Cytogenomic Nomenclature (2016) Reprint of: Cytogenetic and Genome Research 2016*,*Vol. 149, No. 1–2: KARGER, 2016, ISCN 2016.

[14] Tefferi A, Lasho TL, Tischer A, Wassie EA, Finke CM, Belachew AA (2014). The prognostic advantage of calreticulin mutations in myelofibrosis might be confined to type 1 or type 1-like CALR variants. Blood.

[15] Guglielmelli P, Rotunno G, Fanelli T, Pacilli A, Brogi G, Calabresi L (2015). Validation of the differential prognostic impact of type 1/type 1-like versus type 2/type 2-like CALR mutations in myelofibrosis. Blood Cancer J.

[16] Lasho TL, Finke CM, Tischer A, Pardanani A, Tefferi A. Mayo CALR mutation type classification guide using alpha helix propensity. Am J Hematol. 2018. 10.1002/ajh.25065. 10.1002/ajh.25065 29424450

[17] Vannucchi AM, Lasho TL, Guglielmelli P, Biamonte F, Pardanani A, Pereira A (2013). Mutations and prognosis in primary myelofibrosis. Leukemia.

[18] Tefferi A, Lasho TL, Finke CM, Elala Y, Hanson CA, Ketterling RP (2016). Targeted deep sequencing in primary myelofibrosis. Blood Adv.

[19] Pardanani A, Abdelrahman RA, Finke C, Lasho TT, Begna KH, Al-Kali A (2015). Genetic determinants of response and survival in momelotinib-treated patients with myelofibrosis. Leukemia.

[20] Gangat N, Caramazza D, Vaidya R, George G, Begna K, Schwager S (2011). DIPSS plus: a refined Dynamic International Prognostic Scoring System for primary myelofibrosis that incorporates prognostic information from karyotype, platelet count, and transfusion status. J Clin Oncol.

[21] Kourie HR, Ameye L, Paesmans M, Bron D (2017). Improved survival in patients with CALR1 compared to CALR2 mutated primary myelofibrosis: a meta-analysis. Brit J Haematol.

